# The LINC01929/miR-6875-5p/ADAMTS12 Axis in the ceRNA Network Regulates the Development of Advanced Bladder Cancer

**DOI:** 10.3389/fonc.2022.856560

**Published:** 2022-05-12

**Authors:** YuFeng Xiong, MingRui Pang, Yang Du, Xi Yu, JingPing Yuan, Wen Liu, Lei Wang, XiuHeng Liu

**Affiliations:** ^1^Department of Urology, Renmin Hospital of Wuhan University, Wuhan, China; ^2^Department of Pathology, Renmin Hospital of Wuhan University, Wuhan, China

**Keywords:** advanced bladder cancer, competitive endogenous RNA, ADAMTS12, LINC01929, MiR-6785-5p

## Abstract

Considering its speedy development and extremely low 5-year overall survival rate worldwide, bladder cancer (BCa) is one of the most common and highly malignant tumors. Increasing evidence suggests that protein-coding mRNAs and non-coding RNAs, including long non-coding RNAs (lncRNAs) and micro RNAs (miRNAs), play an essential role in regulating the biological processes of cancer. To investigate the molecular regulation associated with poor prognosis during advanced BCa development, we constructed a competitive endogenous RNA (ceRNA) network. Using transcriptome profiles from The Cancer Genome Atlas and Gene Expression Omnibus databases, we performed differential expression (DE) analysis, weighted gene co-expression network analysis, functional enrichment analysis, survival analysis, prediction of miRNA targeting, and Pearson correlation analysis. Through layers of selection, 8 lncRNAs-28 mRNAs and 8 miRNAs-28 mRNAs pairs shared similar expression patterns, constituting a core ceRNA regulatory network related to the invasion, progression, and metastasis of advanced clinical stage (ACS) BCa. Subsequently, we conducted real time qPCR, western blotting, and immunohistochemistry to validate expression trend bioinformatics analysis on 3, 2, and 3 differentially expressed mRNAs, lncRNAs, and miRNAs, respectively. The most significantly differentially expressed LINC01929, miR-6875-5p and ADAMTS12 were selected for *in vitro* experiments to assess the functional role of the LINC01929/miR-6875-5p/ADAMTS12 axis. RNA pull-down, luciferase assays, and rescue assays were performed to examine the binding of LINC01929 and miR-6875-5p. Increasing trends in COL6A1, CDH11, ADAMTS12, LINC01705, and LINC01929 expression variation were verified as consistent with previous DE analysis results in ACS-BCa, compared with low clinical stage (LCS) BCa. Expression trends in parts of these RNAs, such as hsa-miR-6875-5p, hsa-miR-6784-5p, COL6A1, and CDH11, were measured in accordance with DE analysis in LCS-BCa, compared with normal bladder urothelium. Through experimental validation, the cancer-promoting molecule ADAMST12 was found to play a key role in the development of advanced BCa. Functionally, ADAMTS12 knockdown inhibited the progression of bladder cancer. Overexpression of LINC01929 promoted bladder cancer development, while overexpression of miR-6785-5p inhibited bladder cancer development. Mechanistically, LINC01929 acted as a sponge for miR-6785-5p and partially reversed the role of miR-6785-5p. Our findings provide an elucidation of the molecular mechanism by which advanced bladder cancer highly expressed LINC01929 upregulates ADAMTS12 expression through competitive adsorption of miR-6875-5p. It provides a new target for the prognosis and diagnosis of advanced bladder cancer.

## Introduction

According to the Global Cancer Incidence, Mortality and Prevalence (GLOBOCAN) database, bladder cancer (BCa) is a frequently occurring tumor, with the tenth and ninth highest incidence and mortality, respectively, rates worldwide since 2018; predominately in men from European countries, North America, and Western Asia (1–3). In China, BCa incidence and mortality rates were 4.46 and 1.91 per 100, 000 persons, respectively, in 2016 (4). Recent studies investigating the molecular evidence for BCa mechanisms and pathophysiology have shown that low-grade tumors can arise *via* simple hyperplasia and minimal dysplasia. At the molecular level, these tumors are characterized by a loss of heterozygosity on chromosome 9 and mutations in genes associated with cell proliferation, division, and growth, such as FGFR3, TERT, and PIK3CA. Muscle-invasive bladder cancer (MIBC), with mutations and regions of DNA amplification, shares many molecular features with other solid cancers, particularly the loss-of-function of key tumor suppressors, such as wild-type TP53, RB1, and PTEN, leading to cell cycle checkpoint escape and dysregulation of major signaling pathways; thus facilitating the division of molecular subtypes based on transcriptional features (1). Furthermore, gene-expression based molecular subtypes are not only associated with prognosis, but, as biomarkers for MIBC, are also predictive of response to preservation therapy (5). However, implications of transcriptional molecular mechanism dysregulation in the poor prognosis of MIBC or advanced BCa has not been elucidated.

In recent years, an increasing number of reports have shown that dysregulation of long non-coding RNA (lncRNA) and micro RNA (miRNA) subsets, such as DANCR, GAS5, MALAT1, and miR-200s, plays crucial roles in advanced BCa cell aggressiveness and metastasis *via* the enrichment of messenger RNA (mRNA) mutations, such as in TP53, PD-1, and FGFR3, leading to differential epithelial-mesenchymal transition status (EMT), carcinoma *in situ* scores, histologic features, and survival (6–8). These modulated lncRNAs and miRNAs, including PTEN/PTENP1, UCA1, miR-210, and miR-146a-5p, may be novel predictive molecular biomarkers for the prognosis of advanced BCa involving invasion, progression, and metastasis (9, 10). Additionally, they present promising potential therapeutic opportunities in cancer treatment, including in response to chemoresistance and subsequent BCa recurrence, targeting the TGFβ1/lncRNA-LET/miR-145 axis (11). Parts of prospective, non-invasive liquid biopsy markers, including circulating RNAs and small extracellular vesicles (exosomes, associated with BCa progression *via* lncRNA transmission) (12), are promising for personalized medicine due to their ability to provide multiple global snapshots of primary and metastatic tumors (9). To explore the mechanism of lncRNA and miRNA post-transcriptional regulation, a competitive endogenous RNA (ceRNA) hypothesis emerged suggesting that transcripts (pseudogene, lncRNA, circRNA, and mRNA) with shared miRNA binding sites sponge and impair their activity, thereby modulating gene expression (13); thus providing a novel interpretation for lncRNA and miRNA function in invasive, progressive, and metastatic BCa. Recently, empirical evidence supporting this hypothesis has emerged (14–16). Since hundreds of thousands of miRNA-target interactions have been experimentally validated, there is potential for determining a vast ceRNA network that broadly influences miRNA activity; with its overall crucial role based on expression changes in the post-transcriptional regulation of BCa development using bioinformatics tools to predict ceRNA interactions (13, 17, 18). To investigate the key molecules associated with advanced BCa, as characterized by invasion, progression, and metastasis, we constructed converged core ceRNAs based on the differential merging of co-expressed lncRNA-mRNA and miRNA-mRNA regulatory networks using bioinformatics tools analysis; thus providing novel prospective biomarkers for the prognosis of BCa. Furthermore, we preliminarily verified differential expression (DE) of the ceRNA network at the transcriptional and translational levels. Moreover, we identified the LINC01929/miR-6875-5p/ADAMTS12 regulatory axis that is highly associated with the development of advanced bladder cancer through bioinformatics analysis of lncRNA-miRNA-mRNA regulatory network and clinical sample validation, and elucidated that LINC01929 upregulates miR-6875-5p and upregulates the oncogenic molecule ADAMST12 through sponge adsorption, which in turn promotes the progression, invasion, and metastasis of advanced bladder cancer.

## Materials and Methods

### Patients and Tissue Samples

To screen for BCa invasion, progression, and metastasis associated with lncRNAs, mRNAs, and miRNAs, and to identify correlations among them, we used multidimensional data extracted from The Cancer Genome Atlas (TCGA) and NCBI-GEO (https://www.ncbi.nlm.nih.gov/geo/), collecting 431 and 492 tissue samples, respectively; 6966 lncRNAs, 19474 mRNAs, and 2565 miRNAs. Two BCa types were defined based on the TNM staging system and pathological grade: 1) advanced clinical stage (ACS): patients diagnosed with Stage III or IV, and a high or high with pathological T-stage ≥ pT2 pathological grade; 2) low clinical stage (LCS): patients diagnosed with Stage II or I, and a low pathological grade.

A total of 18 primary BCa patients, who had undergone complete resection surgery without preoperative radiotherapy or chemotherapy before being diagnosed by the same pathologists at the Renmin Hospital of Wuhan University in 2019, were used to experimentally verify bioinformatics predictions. Transcriptional and translational levels of selected genes were experimentally detected in 18 BCa tissue samples classified: 1) ACS-BCa tissue samples (n=8): patients diagnosed with a high pathological grade and invasive, with BCa at a clinical stage ≥ T2 (muscle-invasive and metastatic) (n=4); a high pathological grade and invasive, with BCa at clinical stage TaT1 (non-muscle-invasive) (n=4); 2) paired with normal bladder urothelium tissue samples (n=4); 3) LCS-BCa tissue samples (n=10): patients diagnosed with a low pathological grade and non-invasive, with a TaT1 clinical stage (1, 19, 20). Parts of resected specimens were shifted into liquid nitrogen and stored at -80°C for real time quantitative PCR (qRT-PCR); others were fixed in formalin and embedded in paraffin for immunohistochemistry (IHC). This study was approved by the Renmin Hospital of Wuhan University; written informed consent was obtained from all patients.

### RNA Profiling for DE Analysis

Gene expression quantification (HTSeq-counts) data of lncRNA and mRNA from BCa patient transcriptome profiling were downloaded from the NCI Genomic Data Commons (GDC) data portal using the “TCGAbiolinks” package in R (21). Both mRNA and lncRNA gene IDs were annotated in R using the file “gencode.v30.annotation.gtf” downloaded from GENCODE (https://www.gencodegenes.org) (22, 23). Additionally, series matrix files of circulating miRNA profiles (Series GSE113486) in BCa patients and non-cancer human samples were collected from GEO. Gene IDs of miRNAs were annotated using the GEO2R tool on NCBI-GEO (24).

Using integrated DE and Pathway analysis (iDEP.90, http://bioinformatics.sdstate.edu/idep/) (25), DE analysis was performed between ACS- and LCS-BCa tissue samples [Group 1: Stage III (142 samples) compared with Stages II and I (134 samples); Group 2: Stage IV (136 samples) compared with Stages II and I (134 samples)], BCa (392 samples), and non-cancer tissues (100 samples)) for DE-lncRNAs, DE-mRNAs, and DE-miRNAs. Additionally, DE analysis was performed between BCa (412 samples) and normal bladder urothelium tissue (19 samples) for mRNAs and lncRNAs in the ceRNA network.

### Weighted Gene Co-Expression Network Analysis

A total of 2457 DE-mRNAs and DE-lncRNAs, from 412 patient samples, and 2565 miRNAs, from 392 patient samples, were adopted for the construction of a co-expression network *via* weighted gene co-expression network analysis (WGCNA) using the R package (3.6.0 version) (26). Firstly, to determine whether genes with missing values existed, matrices of DE-lncRNAs, DE-mRNAs, and miRNAs were individually examined *via* the “goodSamplesGenes” function in R. Next, “hclust” and “cutreeStatic” functions in R were used for hierarchical clustering to detect any obvious outliers in the input samples, also removing them. Second, a similarity matrix was obtained by calculating the Pearson’s correlation coefficient between each pair of genes; this was transformed to an adjacency matrix using an appropriate soft-thresholding power β value (mRNA and lncRNA: soft-power = 4, miRNA: soft-power = 5), selected using the network topology analysis function, to perform a scale-free topological fit index. A scale-free fit of R^2 = 0.9 was used as the cut-off to obtain a high-confidence scale-free network. Third, transformation of adjacency was turned into a topological overlap by calculating the pair-wise topological overlap (TO) between genes to obtain co-expression modules using the “cutreeDynamic” function in the “dynamicTreeCut” R package. We selected to merge 75%-correlated modules using the “mergeCloseModules” function in the R package. Lastly, for each module, the module membership measure was defined through connectivity, the correlation between gene expression values and module eigengenes, based on the sum of edge weights. Edges and nodes of network list files were visualized using Cytoscape (3.7.1 version) and exported for further analysis based on module-trait heatmap significance; defined as the relationship between modules and clinical traits of samples. Relationships between modules and clinical traits were tested using the “verboseScatterplot” function, while module eigengene relationships were plotted using the “plotEigengeneNetworks” function, both in the R package.

### Functional Enrichment Analysis

Gene ontology enrichment (GO) analysis was performed, including biological process (BP), cell component (CC), and molecular function (MF), using the WEB-based Gene Set Analysis Toolkit (WebGestalt, http://www.webgestalt.org) (27). Through the Kyoto Encyclopedia of Genes and Genomes (KEGG), a pathway analysis was performed to explore which genes were involved in signaling pathways using the ClueGO App in Cytoscape (28, 29). In addition, relationships among enriched clusters from GO and KEGG pathway analyses were visualized *via* Venn diagrams in R and Cytoscape, respectively. Furthermore, hub genes were used to perform functional enrichment analysis to explore the protein-protein-interaction (PPI) network *via* the String database (https://string-db.org) (30). The cut-off value was 0.05, and the P-value was 0.01.

### Survival Analysis

Using the Xena Functional Genomics Explorer from the University of California Santa Cruz (UCSC) Xena database (https://xenabrowser.net/datapages/), Kaplan-Meier survival curves were generated to evaluate the prognostic value of selected mRNAs and lncRNAs *via* overall survival (31). A log-rank test was performed, and a P-value < 0.05 was set as statistically significant for screening. Furthermore, to plot K-M graphs using GraphPad Prism 8.0, patient clinical survival data and each RNA expression profile were downloaded from the GDC TCGA BLCA database on the UCSC Xena database. BCa patients were divided into two groups according to median mRNAs or lncRNAs expression, and the P-value was calculated.

### Construction of the lncRNA-miRNA-mRNA Network

Predicting which mRNAs were targeted by miRNAs was performed through the sharing of miRNA IDs on miRWalk and miRTarbase databases. To verify this prediction, miRNA and mRNA expression quantification data from 414 patient samples were collected from the GDC TCGA BLCA database on UCSC Xena, to undergo Pearson’s correlation analysis using the “corr.test” function in “Hmisc” of the R package. Furthermore, statistical significance was set at *p* < 0.05 to determine significant miRNAs-mRNAs and mRNAs-mRNAs pairs; a correlation coefficient matrix was established and visualized with selected miRNAs-mRNAs and mRNAs-mRNAs pairs using the “corrplot.mixed” function in “corrplot” of the R package. Lastly, lncRNA-miRNA-mRNA network construction was based on the weights of all edges connecting to module eigengenes, and the Pearson’s correlation coefficient of miRNA-mRNA interaction pairs visualized on Cytoscape.

### Cell Lines and Cultures

Three BCa cell lines (T24, EJ, and 5637) and a human immortalized uroepithelial cell line (SV-HUC-1) were purchased from the American-Type Culture Collection. Cancer cell lines were cultured in RPMI-1640 medium (HyClone, China), while the SV-HUC-1 cell line was cultured in F-12K medium (HyClone, China); both media were supplemented with 10% fetal bovine serum (Gibco, Australia) and 1% penicillin G sodium/streptomycin sulfate at 37°C in a humidified CO_2_ (5%) atmosphere.

### qRT-PCR

Total RNA was isolated from the liquid nitrogen tissue samples of 16 BCa patients using the KZ-II grinder TRIzol reagent (Cat. abs9331-100ml, Absin, China) in an RNase-free atmosphere. The quality of total RNA was evaluated using a NanoDrop (Cat. #N60, Implen, Germany). Next, in a 20 ul total reaction volume using a PrimeScript™ RT reagent Kit with gDNA Eraser (Takara, Japan), the cDNA of mRNAs and lncRNAs was synthesized from the < 1000 ng of total extracted RNA, which was then amplified in a 25 ul total reaction volume with TB Green^®^ Premix Ex Taq™ II (Tli RNaseH Plus, Takara) using a CFX96 Real-Time PCR Detection System. Additionally, miRNA cDNA was synthesized from 250 ng < total RNA < 800ng in a 10 ul total reaction volume, and amplified in a 25 ul total reaction volume using Mir-X™ miRNA First- Strand Synthesis and TB Green™ qRT-PCR (Takara), according to the manufacturers protocol. The housekeeping gene, glyceraldehyde-3-phosphate dehydrogenase (GAPDH), was used as an internal control to calculate the comparative quantification of gene expression levels *via* the 2-ΔΔCt method. All primers, the sequences of which are listed below, were synthesized by Sangon Biotech (Shanghai).

COL6A1 (ENSG00000142156.13):
 forward, 5’-TCGTGGACAAAGTCAAGTCC-3’;
 reverse, 5’-GATGATCTCCACCTCGTCAC-3’.
CDH11 (ENSG00000140937.12):
 forward, 5’-ATCCTCGCCTGCATCGTCATTC-3
 reverse, 5’-CAGCTGGAAGCCCAGATAAAGCA-3’.
ADAMTS12 (ENSG00000151388.9):
forward, 5’-TGGGAACCTCTCCCATGTTAAG-3
 reverse, 5’-CTCAACCAGTGGATGCTTCTTC-3’.
LINC01705 (ENSG00000232679.1):
forward, 5’-TCCTGCATCAGAGCCACTCA-3
 reverse, 5’-GCACTGACCATGACAACACAGG-3’.
LINC01929 (ENSG00000267013.4):
forward, 5’-CTGAATCCTATTCTGGCCCATCG-3’;
 reverse, 5’-CTCTCTGCTCCTCTTCTCACTTC-3’.
hsa-miR-128-2: 5’-TCACAGTGAACCGGTCTC-3’.
hsa-miR-6784-5p: 5’-GCCGGGGCTTTGGGTGAGGG-3’.
hsa-miR-6875-5p: 5’-TGAGGGACCCAGGACAGGAGA-3’.

### Western Blotting (WB)

Protein lysates were prepared from BCa and SV-HUC-1 cell lines using a RIPA buffer solution supplemented with a 1% volume of phosphatase inhibitor (Sigma-Aldrich, USA) and 1% protease inhibitor. Lysates were then electro-transferred into 8% SDS-PAGE, and then onto polyvinylidene difluoride (PVDF) membranes (Millipore, USA), before blocking with Tris-buffered saline with 0.05% Tween-20 (TBS-T) containing 5% non-fat milk for 1 h. PVDF membranes were incubated overnight with primary antibody (COL6A1, CDH11, ADAMTS12, GAPDH: 1/1000) at 4°C, followed by incubation with a secondary antibody for 1 h at room temperature; bands were exposed using an enhanced chemiluminescence (ECL) kit (Millipore, USA) on a Bio-Rad ChemiDoe XRS+ Imaging System (Bio-Rad, USA).

### IHC

Paraffin-embedded sections from of 22 BCa patients tissue samples were deparaffinized atin 62°C for 1 h, following, and dehydrationed with xylene, 100% ethanol, and followed by decreased concentrations of ethanol. Antigen retrieval was performed in sodium citrate solution with by boiling for 10 min and blockinged with peroxidase. Next, sections were incubated with the primary antibody (COL6A1 1/100, CDH11 1/200, ADAMTS12 1/200, Aabcam, USA) atin 4°C overnight, and then secondary antibody in at roommate temperature for 0.5 h. Sections were stained with 3, 3-diaminobenzidine (DAB) for minutes while operating in an optical microscope, followed by and counterstaininged with Hematoxylin for 15 s. The images were acquired using with an upright microscope system (Nikon, JAPAN). The final immunostaining score was measured by using the mean optical density as analyzed by in Image-Ppro Pplus (version 6.0). The mean density estimationor formula was as follows:


$$mean\;density=\;\frac{IOD}{AOI}$$

where IOD denotes the sum of integrated optical density and AOI denotes the sum of areas of interesting. Subsequently, each the value of mean density value of for each the immunohistochemical photo image was calculated for statistical differences among groups.

### Small Interfering RNA (siRNA) Transfection

Cells were transfected with siRNA specific for ADAMTS12 for 48 h, while non-targeting siRNA was used as a negative control. All siRNAs were used at a concentration of 100 nM. After 6 h of transfection, cells were incubated in DMEM/F12 containing 0.2% FBS for 48 h. RT-PCR was performed to confirm the effect of siRNA transfection.

### Transfections and Selection of BCa Cell Lines With Stable Overexpression of LINC01929, miR-6875-5p and ADAMTS12

The full sequences of LINC01929, miR-6875-5p and ADAMTS12 were inserted into a lentiviral vector to construct an overexpression plasmid (Vigenebio, China). BCa cells (1 × 10 ^5^) were first inoculated in 6-well plates, and the medium was removed when they grew to approximately 50% confluence and fresh medium containing lentiviral particles carrying LINC01929, miR-6875-5p and ADAMTS12 cDNAs or a negative control was added according to the manufacturer’s instructions. Cells were then incubated at 37°C and 5% CO_2_ for 18 h. After changing the medium and continuing transfection for 72 h, medium containing the appropriate concentration of puromycin (Sigma, USA) was added to kill any untransfected cells. Surviving cell clones were selected and amplified. The empty vector was used as a negative control (pcDNA-vector). qRT-PCR analysis was used to assess the efficiency of infection.

### CCK-8-Based Cell Viability Assay

Cell viability was assessed using the Cell Counting Kit-8 assay (Beyotime Biotechnology, #C0037) according to the manufacturer’s instructions. Briefly, BCa cells were inoculated into 96-well plates, after which they were pre-cultured in an incubator for 24 h (37°C, 5% CO_2_) to allow cell apposition. After different treatments, 10 uL of CCK-8 solution was added to each well and incubated in the incubator for 2 h. The absorbance was measured at 450 nm using a microplate reader (Molecular Devices, USA). Cell growth curves were plotted according to the results of each experiment.

### Transwell Migration Assay

After transfection, 8 × 10^4^ cells were suspended in 200 μl of serum-free medium and added to the upper chamber, while 500 μl of 20% FBS medium was added to the lower chamber to induce cell migration. after 72 h, the remaining cells in the upper chamber were removed. Cells that migrated to the other side of the membrane were fixed with 4% PFA for 30 min and finally stained with 0.1% crystalline violet for 4 h.

### Flow Cytometry

Apoptosis was assessed by flow cytometry using the Annexin-V FITC Apoptosis Detection Kit I (BD Biosciences, USA) according to the manufacturer’s instructions. Briefly, BCa cells were washed twice with PBS and stained with a binding buffer containing 5 μL Annexin V-FITC in 10 μL PI for 5 min under light-proof and room temperature conditions. Apoptotic cells were detected by FACS flow cytometry (BD, Germany).

### Tumor Cell Colony Formation Assay

We assessed the clonogenic function of tumor cells using a colony formation assay. Cells from different treatment groups were inoculated at 1×10^3^ cells/well in 6-well plates and grown for 10 days. Finally, the cells were fixed with 4% paraformaldehyde (PFA) and stained with 0.1% crystalline violet.

### Luciferase Activity Assay

One day before transfection, 1 × 10^5^ T24 cells were seeded in 6-well plates. Cells carrying wild-type or mutant LINC01929 plasmids were then cotransfected with miR-6875-5p mimics or NC mimics into pre-seeded cells; wild-type or mutant ADAMTS12 was cotransfected with miR-6875-5p mimics or NC mimics into pre-seeded cells. The transfected cells were then seeded into 96-well plates at a density of 1 × 104 cells per well, and luciferase activity was measured according to the instructions of Dual-Luciferase Reporter Assay (Promega, USA).

### RNA Pull-Down Assay

To prepare streptavidin magnetic beads, the beads were washed three times with RNase-free lysis buffer, blocked with 10 μg/μL BSA and yeast tRNA on a rotator at 4°C for 3 h. The blocked magnetic beads were incubated with biotinylated miR-6875-5p probe or oligo probe for 2 h at 25°C. Afterwards, the prepared probe and cell lysate were incubated overnight at 4°C and 50 μL of cell lysate was collected as input. Pull-down complexes were harvested and detected by quantitative real-time PCR (qRT-PCR).

### Statistical Analysis

Statistical analyses were performed using R Studio (R version 3.6.0), GraphPad Prism 8.3.1, and relevant data-analyzing websites. Unpaired, independent sample t tests were used to compare two groups. The false discovery rate (FDR) was calculated to correct the P-value associated with RNA-sequencing analysis. All independent experimental tests were performed more than 3 times, and P-value < 0.05 was considered statistically significant.

## Results

### Identification of DE lncRNAs-mRNAs and miRNAs Correlated With ACS-BCa

**Figure 1:**
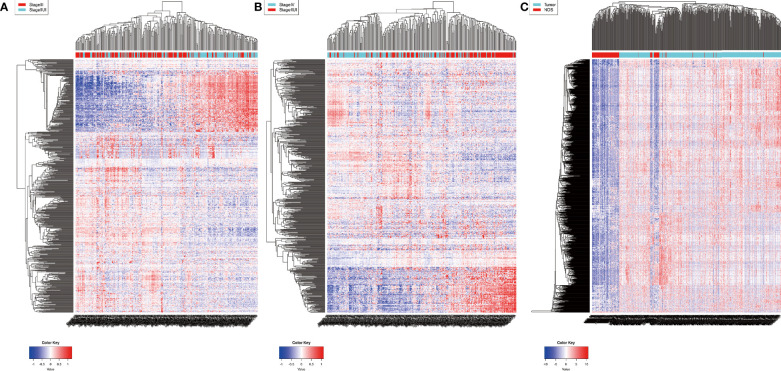
Identification of differentially expressed RNAs in bladder cancer. heatmap **(A, B)** 2076 mRNAs and 381 lncRNAs were differentially expressed between ACS-BCa and LCA-BCa samples (429 and 501 upregulated mRNAs, 59 and 38 upregulated lncRNAs, and, 138 and 101 downregulated mRNAs, 17 and 23 down-regulated lncRNAs) (P < 0.05, |fold change (FC)| > 2.0); heatmap **(C)**1696 miRNAs were differentially expressed in BCa compared to non-cancerous tissue samples (1593 up-regulated and 103 down-regulated) (P < 0.01, |FC| > 2.0).

Remarkably, 2076 mRNAs and 381 lncRNAs were differentially expressed between ACS-BCa and LCA-BCa samples (*P* < 0.05, |fold change (FC)| > 2.0) from TCGA, respectively containing 429 and 501 up-regulated mRNAs, and 59 and 38 upregulated lncRNAs, as well as, 138 and 101 downregulated mRNAs, and 17 and 23 downregulated lncRNAs (**Figures 1A, B**). Similarly, 1696 miRNAs were differentially expressed between BCa and non-cancer tissue samples (*P* < 0.01, |FC| > 2.0) from GEO, including 1593 upregulated, and 103 downregulated, miRNAs (**Figure 1C**).

**Figure 2:**
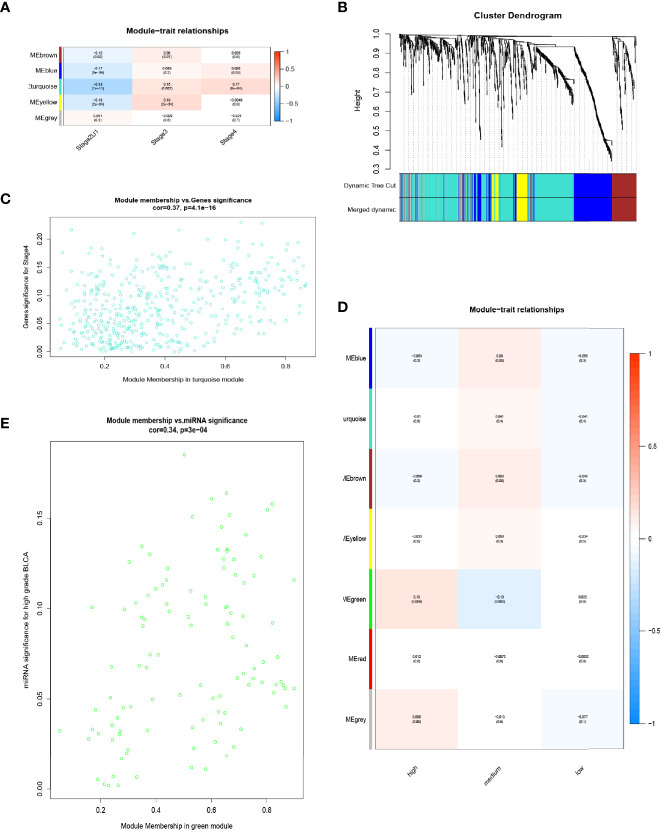
WGCNA was used for DE lncRNAs-mRNAs and miRNAs. **(A)** Correlation between RNA modules and clinical stage of samples. **(B)** Dynamic tree-cutting and merging dynamic methods to identify 14 RNA modules. **(C)** Sucessive analysis of brown mRNA-lncRNA modules with stage IV (cor. = 0.37, P = 4.1e-16). **(D)** Expression data of miRNAs from GEO-BCa were classified into three clinical features for WGCNA: high grade (high pathological grade with pathological T-stage ≥ pT2), medium grade (high pathological grade with pathological T-stage < pT2) and low grade (low pathological grade). **(E)** Correlation between green module (108 miRNAs) and high grade.

To determine a correlation between differentially expressed RNA and ACS-specific BCa, WGCNA was performed using the expression matrix of DE-mRNAs and DE-lncRNAs. Based on the clinical information of patients from TCGA-BCa, clinical traits were divided into three groups, including Stage IV, Stage III, and Stage II&I, prior to identifying 14 RNA modules using dynamic tree cutting and merged dynamic methods (**Figure 2B**). Next, correlations between RNA modules and clinical stage of the samples were analyzed (**Figure 2A**). The turquoise module with the highest positive or lowest negative scores was found to positively correlate with Stage III and Stage IV or passively Stage II&I, respectively (*P* < 0.05), and consisted of 395 DE-mRNAs and 56 DE-lncRNAs, which were selected to perform successive analysis. A significant positive correlation (cor. = 0.37, *P* = 4.1e-16) was found between brown mRNA-lncRNA modules and Stage IV (**Figure 2C**). Furthermore, the expression data of miRNAs from GEO-BCa were classified into three clinical traits to perform WGCNA: high grade (a high pathological grade with a pathological T-stage ≥ pT2), medium grade (a high pathological grade with a pathological T-stage < pT2), and low grade (a low pathological grade). The results indicated that the green module (108 miRNAs) significantly correlated with high grade (cor. = 0.34, *p* = 3e-04) (**Figures 2D, E**). Moreover, 108 miRNAs intersected with the 1696 differentially expressed miRNAs for acquisition of 11 miRNAs through analysis of miRNA expression quantification on the UCSC Xena browser. The results indicated that 395 DE-mRNAs, 56 DE-lncRNAs, and 11 DE-miRNAs may be the key to constructing a ceRNA network that highlights vital roles in the progression, invasion and metastasis of LCS-BCa developing into ACS.

### Screening for Genes Characterized by Functional Enrichment Associated With BCa Invasion, Progression, Metastasis and Poor Prognosis

**Figure 3:**
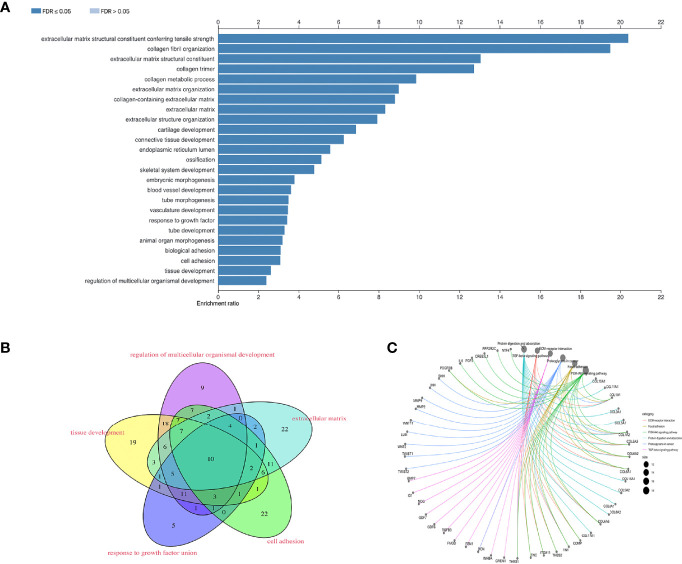
To screen for functionally enriched gene signatures associated with BCa invasion, progression, metastasis and poor prognosis. **(A)** The 395 co-expressed DE-mRNA enriched GO terms are mainly (FDR < 0.05) collagen fiber organization, extracellular matrix organization, vascular development, response to growth factors, bioadhesion, and tissue development. **(B)** Enrichment of biological processes such as extracellular matrix, tissue development, response to growth factors, cell adhesion and developmental regulation of multicellular organisms. **(C)** KEGG analysis of 395 co-expressed DE-genes.

GO terms in which the 395 co-expressed DE-mRNAs, based on the ACS-specific gene module, were enriched were predominantly (FDR < 0.05) collagen fibril organization, extracellular matrix organization, blood vessel development, response to growth factor, biological adhesion, and tissue development (**Figure 3A**). To further identify genes functionally related to BCa invasion, progression, and metastasis, enrichment of the following biological processes was selected and unified: extracellular matrix, tissue development, response to growth factor, cell adhesion, and regulation of multicellular organismal development (**Figure 3B**); consequently, 188 mRNAs were obtained. Additionally, through KEGG analysis, we found the 395 co-expressed DE-genes to be significantly (*p* < 1e−10) enriched in ECM-receptor interaction, focal adhesion, and TGFβ and PI-3 kinase (PI3K)/AKT signaling pathways (**Figure 3C**). Interestingly, recent reports have shown that cancer-associated fibroblasts (CAFs) play a role in producing the extracellular matrix (ECM) structure, while cancer is associated with fibroblasts at all stages of disease progression, including metastasis (32, 33). Furthermore, dysregulation of TGFβ responsiveness and its downstream signaling pathways contributes to cancer invasion, progression, and metastasis (34). BCa cells trigger fibroblast differentiation into CAFs *via* exosome-mediated TGFβ transfer and SMAD pathway activation (35). Regulation of the PI3K/Akt signaling pathway is essential for maintaining the integrity of fundamental cellular processes, cell growth, survival, death and metabolism, with dysregulation of this pathway implicated in the development and progression of cancers (10).

**Figure 4:**
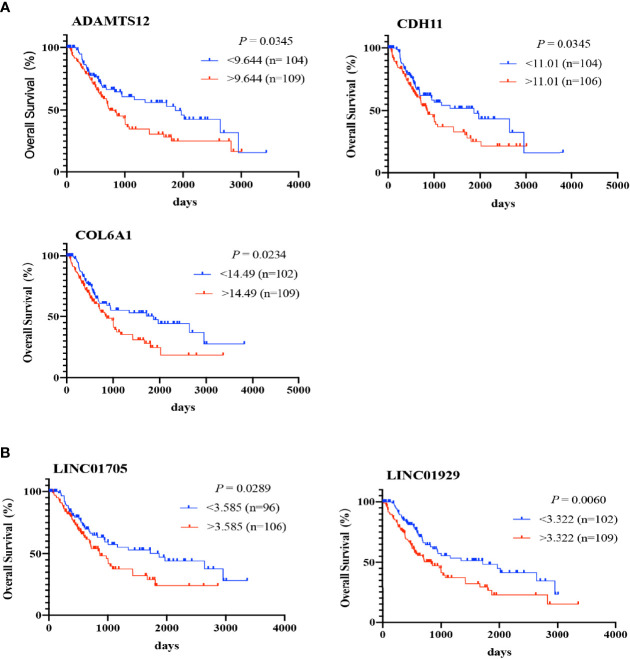
Survival analysis performed for 8 core lncRNAs and 28 core mRNAs. The Kaplan-Meier curve shows the percent survival rate for BCa patients based on RNA expression in BCa tissue samples. The P-value is calculated using the log-rank test (p < 0.05) **(A, B)**.

188 and 56 significantly co-expressed DE-mRNAs and lncRNAs were performed for survival analysis to identify RNAs correlated with the dysregulation-associated poor prognosis of BCa patients from UCSC Xena. Using Xena Functional Genomics Explorer, 6 lncRNAs and 83 mRNAs were screened out; reaching statistical significance (*p* < 0.05). A fraction of K-M curves is shown in **Figures 4A, B**. As a result, consistent with results of DE analysis, all of highly expressed RNAs contributed to poor prognosis. They were identified as oncogenes, as high expression levels of these RNAs correlated with short survival time during BCa development.

### Prediction of miRNA-mRNA Pairs and RNA-Based Signature Prognostic Model

**Figure 5:**
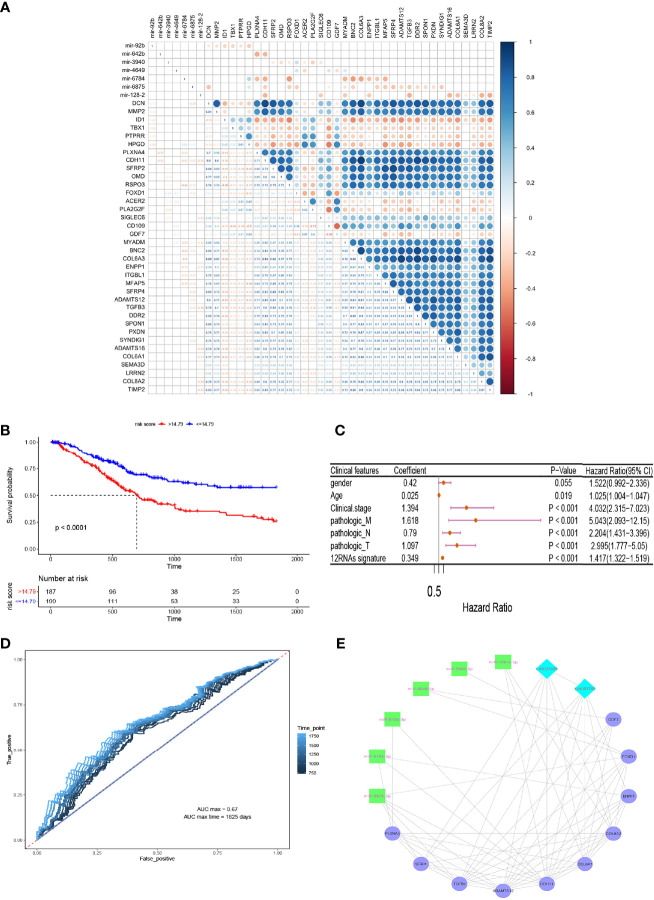
Prediction of miRNA-mRNA pairs and RNA-based signature prognostic models. **(A)** Pearson correlation analysis to establish a correlation coefficient matrix. **(B)** A RNA signature model for survival risk analysis. **(C)** univariate Cox regression analysis exhibited that the twelve-RNA signature was evidently positively correlated with overall survival of BCa patients. **(D)** 2 to 5 years dependent ROC curves. **(E)** Core lncRNA-miRNA-mRNA triple-regulatory co-expression network.

Thousands of target genes of the above-mentioned 11 aberrantly co-expressed miRNAs from GEO were predicted. Next, candidate target mRNAs were intersected with 83 survival-analyzed mRNAs, and 141 miRNA-mRNA interaction pairs containing 8 miRNAs and 54 mRNAs were collected. Furthermore, to evaluate the relationships between miRNA-mRNA pairs and mRNA-mRNA interaction pairs, a correlation coefficient matrix was established *via* a Pearson’s correlation analysis (**Figure 5A**). Notably, all of the miRNAs were negatively correlated with the majority of mRNAs, implying that expression of the relevant mRNAs was likely downregulated by these miRNAs (max. |cor.| = 0.5). Finally, 58 miRNA-mRNA pairs containing 7 miRNAs and 36 mRNAs were obtained. Hence, 6 survival-analyzed lncRNAs and 36 mRNAs were subjected to perform Univariate and multivariate Cox regression analysis to construct a RNA signature model for survival risk analysis.

The results showed that highly co-expressed 10 mRNAs and 2 lncRNAs were associated with poor prognosis of BCa (Hazard Ratio HR>1, *P* < 0.05)(**Figure 5B**). Consequently, a predictive twelve-RNA signature model including 10 mRNAs and 2 lncRNAs was established based on the summation of the product of each RNA and its coeffcient in multivariate Cox regression as follows (36): twelve-RNA signature risk score = (0.2242588 × expression of LINC01705) + (0.2085348 × expression of LINC01929) + (0.4987447 × expression of CDH11) + (0.6365046 × expression of ADAMTS12) + (0.3202686 × expression of COL6A1) + (0.204348 × expression of PLXNA4) + (0.1172562 × expression of FOXD1) + (0.200191 × expression of GDF7) + (0.2104752× expression of ENPP1) + (0.321133 × expression of SFRP4) + (-0.4043371× expression of TGFB3) + (-0.5691683 × expression of COL8A2). To investigate the independence of nine-RNA signature considering other clinical factors related to BCa prognosis, such as gender, age, clinical stage, distant metastasis (M), Lymph-node status (N) and T stage, univariate Cox regression analysis exhibited that the twelve-RNA signature was evidently positively correlated with overall survival of BCa patients (coefficient = 0.349, hazard ratio HR = 1.42, confidence interval 95% CI = 1.32–1.52, *P* = 2.00e-16)(**Figure 5C**). Furthermore, to evaluate the accuracy and ability of twelve-RNA signature model for predicting poor prognosis of BCa, 2 to5 years dependent ROC curves were plotted, which showed the max AUC value of 5 years (AUC max time) dependent ROC curve (AUC = 0.67)(**Figure 5D**). These results indicated that the twelve RNAs have potential prognostic value and might be used to establish a novel RNA-based model for BCa patients survival prediction.

### Construction of the Critical Co-Expression lncRNA-miRNA-mRNA Network

To explore the relationships among above 2 lncRNAs and 10 mRNAs correlated with 6 miRNAs, which were intensely associated with invasion, progression, metastasis, and poor prognosis of BCa, and to better understand the regulatory mechanism, the core lncRNA-miRNA-mRNA triple regulatory co-expression network was established based on mRNA connectivity (**Figure 5E**). To verify underlying biomarkers value of bioinformatics analysis, we performed expression levels verification and cellular functions and the mechanism experiments for acquired RNA regulatory network.

### Transcriptional and Translational Level Preliminary Expression Variation Trend Verification in a Part of the Network

**Figure 6:**
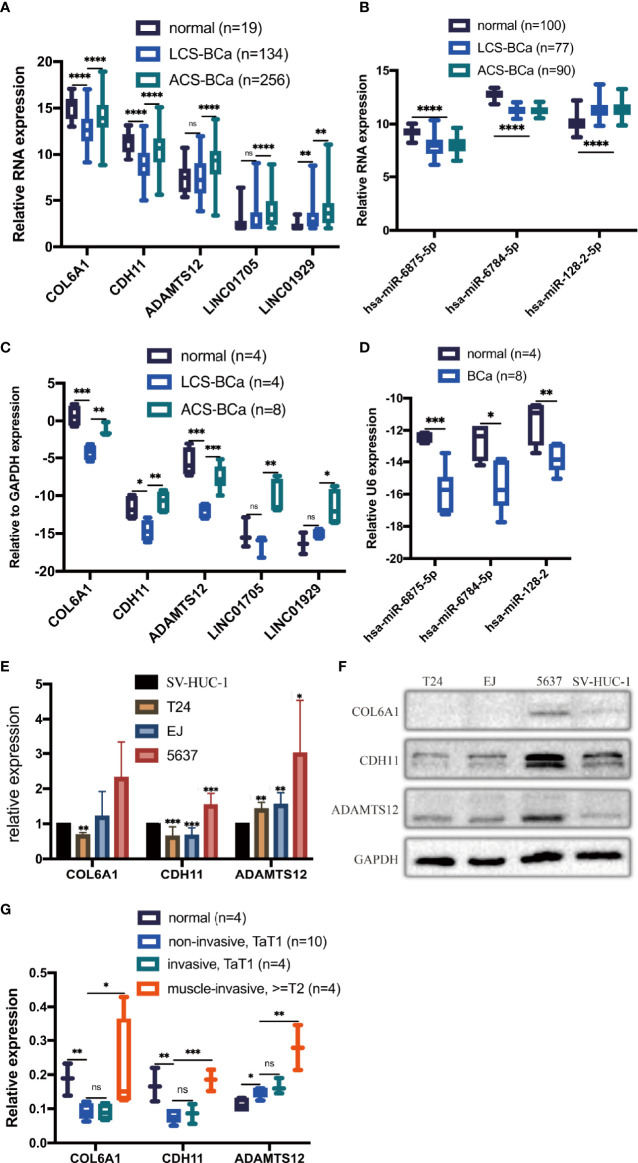
Expression trends of 2 chosen lncRNAs, 3 mRNAs, and 3 miRNAs were verified using experiments. Relative expression of mRNAs, lncRNAs, and miRNAs in BCa tissues was analyzed using DE analysis of bioinformatics data **(A, B)**. Relative expression of mRNAs, lncRNAs, and miRNAs in BCa patient tissues was measured using qRT-PCR **(C, D)**. Relative expression of 3 proteins extracted from BCa cell lines and an immortalized uroepithelial cell line was measured using WB **(E, F)**. Relative expression of 3 proteins in BCa patient tissues was measured using IHC **(G)**. *P < 0.05; **P < 0.01; ***P < 0.005; ****P < 0.001. The extended form of “ns” is “no significance”, which means that there is no statistical difference between the two groups.

To preliminarily demonstrate the feasibility and value of the constructed RNA network, we selected the RNAs located in the core of the network, with the strongest correlations and the most contact nodes: 3 genes (COL6A1, CDH11, and ADAMTS12), 2 lncRNAs (LINC01705 and LINC01929), and 3 miRNAs (hsa-miR-6875-5p, hsa-miR-6784-5p, and hsa-miR-128-2). These RNAs were used to examine expression levels in 22 BCa patient tissue samples, BCa cell lines (T24, EJ, and 5637), and an immortalized uroepithelial cell line (SV-HUC-1). According to the abovementioned DE analysis, COL6A1, CDH11, ADAMTS12, LINC01705 and LINC01929 were upregulated by a 2.6-, 2.8-, 3.2-, 2.9- and 2.5-fold change, respectively, in ACS-BCa compared with LCS-BCa tissue. Whereas, COL6A1 and CDH11 were downregulated by a 3.0- and 2.6-fold change, respectively, and LINC01705 and LINC01929 was up-regulated by a 3.0- and 4.5-fold change, while ADAMTS12 showed a lack of expression differences in BCa compared with normal tissue. In addition, hsa-miR-6784-5p and hsa-miR-6875-5p were downregulated 2.9- and 2.6-fold, respectively, while hsa-miR-128-2-5p was upregulated 2.2-fold in BCa tissue compared with normal tissue (**Figures 6A, B**).

qRT-PCR results showed that the RNA levels of COL6A1, CDH11, and ADAMTS12 significantly increased in ACS-BCa compared with LCS-BCa tissue, also decreasing in LCS-BCa tissue compared with normal uroepithelial tissue; LINC01705 and LINC01929 significantly increased in ACS-BCa compared with LCS-BCa tissue. In contrast, hsa-miR-6875-5p, hsa-miR-6784-5p, and hsa-miR-128-2 decreased in BCa tissue compared with normal uroepithelial tissue. In summary, aside from the reverse tendency of hsa-miR-128-2 and the non-significant expression variation of ADAMTS12, LINC01705 and LINC01929 in LCS-BCa compared with normal tissue, the expression tendency and pattern of gene RNA levels were all consistent with DE analysis (**Figures 6C, D**).

WB results showed that, except for the non-significant variation of COL6A1 in EJ and 5637 and the opposite expression variation trend of CDH11 in the 5637, COL6A1, CDH11, and ADAMST12 protein expression significantly decreased in T24 and EJ compared with the SV-HUC-1; ADAMTS12 protein expression increased significantly in 5637 (**Figures 6E, F**). These results were in agreement with the results of qRT-PCR.

**Figure 7:**
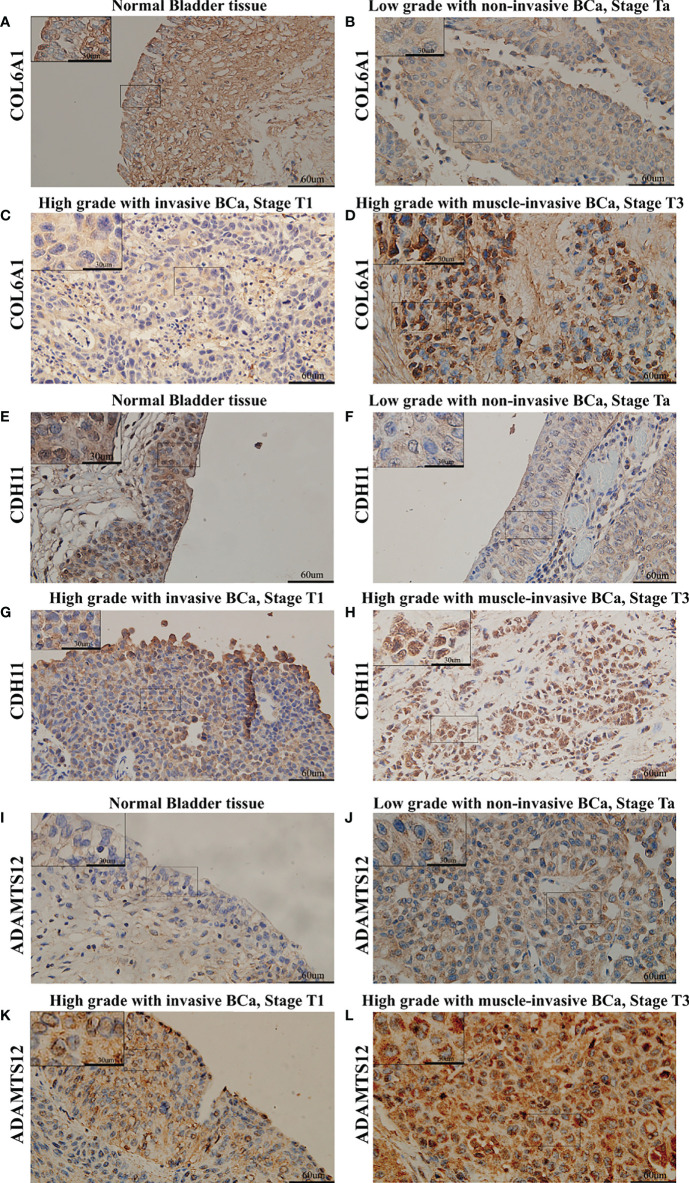
Expression of COL6A1, CDH11, and ADAMTS12 was measured using IHC in BCa tissues divided into different groups. **(A)** Expression of COL6A1 in normal bladder tissue. **(B)** Expression of COL6A1 in low grade with non-invasive BCa, Stage Ta. **(C)** Expression of COL6A1 in high grade with invasive BCa, Stage T1. **(D)** Expression of COL6A1 in high grade with muscle-invasive BCa, Stage T3. **(E)** Expression of CDH11 in normal bladder tissue. **(F)** Expression of CDH11 in low grade with non-invasive BCa, Stage Ta. **(G)** Expression of CDH11 in high grade with invasive BCa, Stage T1. **(H)** Expression of CDH11 in high grade with muscle-invasive BCa, Stage T3. **(I)** Expression of ADAMTS12 in normal bladder tissue. **(J)** Expression of ADAMTS12 in low grade with non-invasive BCa, Stage Ta. **(K)** Expression of ADAMTS12 in high grade with invasive BCa, Stage T1. **(L)** Expression of ADAMTS12 in high grade with muscle-invasive BCa, Stage T3.

IHC results showed that COL6A1, CDH11, and ADAMTS12 protein expression increased in ACS-BCa compared with LCS-BCa; COL6A1 and CDH11 protein expression decreased, while ADAMTS12 increased, in LCS-BCa compared with normal bladder urothelium tissue (**Figures 6G**, **7A–L**). These results suggest that RNAs and proteins expression tendency and pattern in ACS-BCa compared with LCS-BCa, were consistent with qRT-PCR and DE bioinformatics analyses.

### Effects of LINC01929, miR-6875-5p and ADAMTS12 on Bladder Cancer Cells

**Figure 8:**
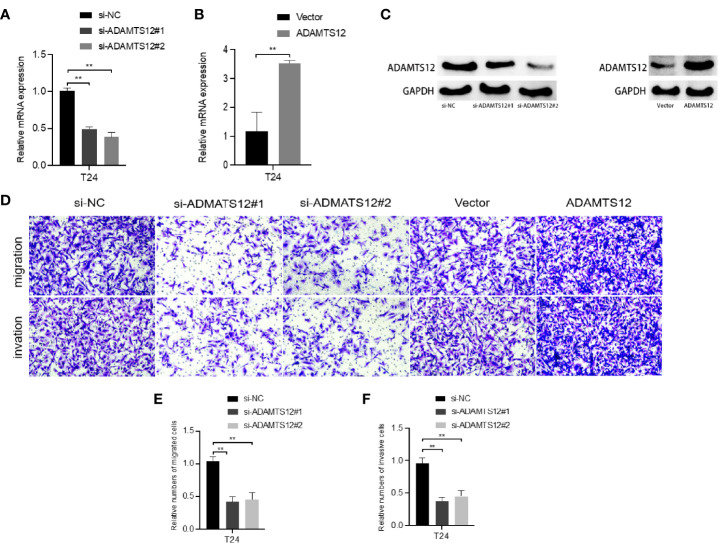
Effect of knockdown or overexpression of ADAMTS12 on T24 cells. **(A)** Validation of the knockdown effect of ADAMTS12 at the mRNA level in T24 cells. **(B)** Validation of the effect of ADAMTS12 overexpression at the mRNA level in T24 cells. **(C)** Validation of knockdown versus overexpression of ADAMTS12 protein levels. Transwell assays **(D)** show that knockdown of ADAMTS12 attenuates cell invasion. **(E)** Relative numbers of migrated cells. **(F)** Relative numbers of invasive cells. **P < 0.01.

The current data suggest that ADAMTS12 is differentially expressed in advanced bladder cancer compared to normal tissues. Therefore, we selected ADAMTS12 for further *in vitro* functional assays to verify whether it has an effect on the progression and invasion of bladder cancer. We knocked down ADAMTS12 with siRNA. qRT-PCR (**Figure 8A**) data showed that RNA expression was significantly downregulated in the si-ADAMTS12 group compared to the si-NC group, demonstrating that we successfully knocked down ADAMTS12. After that, ADAMTS12 was overexpressed with plasmid, and qRT-PCR (**Figure 8B**) data showed that compared to the Vector group Western blotting experiments (**Figure 8C**) also further confirmed the knockdown and overexpression effects. Transwell assays (**Figures 8D–F**) confirmed that knockdown of ADAMTS12 inhibited invasion of T24 cells while overexpression of ADAMTS12 enhanced the invasion of T24 cells.

**Figure 9:**
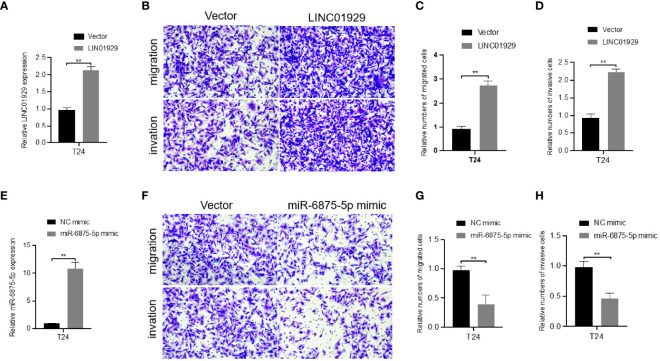
Effect of overexpression of LINC01929 and miR-6875-5p on T24 cells. **(A)** Validation of the effect of LINC01929 overexpression at the RNA level in T24 cells. Transwell assays **(B)** show that overexpression of LINC01929 enhances cell invasion. **(E)** Validation of the overexpression effect of miR-6875-5p at the RNA level in T24 cells. Transwell assays **(F)** show that overexpression of miR-6875-5p attenuates cell invasion. **(C, G)** Relative numbers of migrated cells. **(D, H)** Relative numbers of invasive cells. **P < 0.01.

Since LINC01929 and miR-6785-5p showed the strongest correlation with ADAMTS12 in bioinformatics analysis, and the validation of differential expression experiments showed high consistency of their expression patterns, we selected them for subsequent functional experiments. The first step was to overexpress LINC01929 and miR-6785-5p by plasmid. qRT-PCR (**Figures 9A, E**) data demonstrated that we successfully overexpressed LINC01929 and miR-6785-5p. transwell assays showed that overexpression of LINC01929 enhanced the invasive ability of T24 cells (**Figures 9B–D**), while overexpression of miR-6785-5p had the opposite effect (**Figures 9F–H**).

### LINC01929 Antagonizes the Inhibitory Effect of miR-6875-5p on ADAMTS12 Expression Through Sponge Adsorption

**Figure 10:**
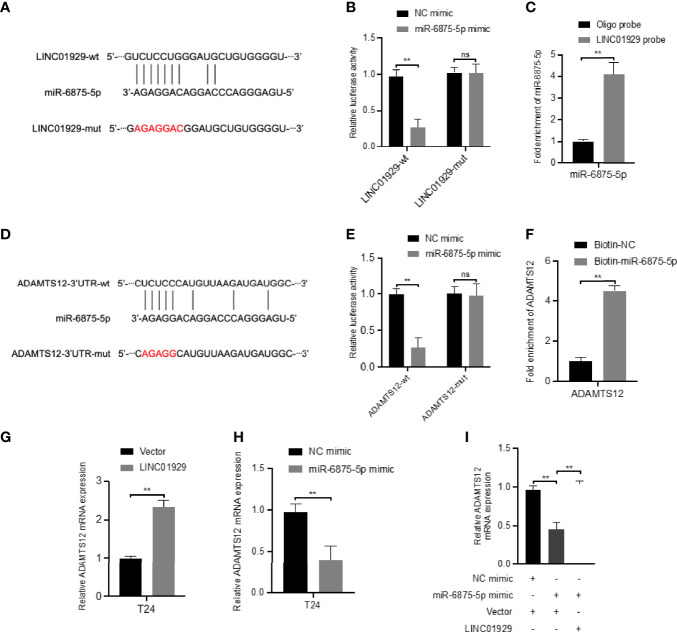
LINC01929 antagonizes miR-6875-5p mediated repression of ADAMTS12 expression. **(A)** Schematic illustration showed the alignment of miR-6875-5p with LINC01929 and the red portion indicated the mutagenesis nucleotides. **(B)** Dual luciferase reporter assays showed the luciferase activity of wild type or mutant LINC01929 following co-transfection with miR-6875-5p mimic or control mimic. Relative firefly luciferase expression was normalized to that of Renilla luciferase. **(C)** RNA pulldown assays revealed that miR-6875-5p directly interact with LINC01929 in T24. **(D)** Schematic illustration showed the alignment of miR-6875-5p with ADAMTS12 and the red portion indicated the mutagenesis nucleotides. **(E)** Dual luciferase reporter assays showed the luciferase activity of wild type or mutant ADAMTS12 following co-transfection with miR-6875-5p mimic or control mimic. **(F)** RNA pulldown assays showed that ADAMTS12 was captured by biotinylated miR-6875-5p. **(G, H)** The effect of LIN01929 and miR-6875-5p overexpression on ADAMTS12 mRNA expression in T24 cells was assessed by qRT-PCR. **(I)** qRT-PCR analyzed the effect of overexpressed LIN01929 on miR-4875-5p overexpression-induced ADAMTS12 expression in T24 cells. **P < 0.01. The extended form of “ns” is “no significance”, which means that there is no statistical difference between the two groups.

**Figure 11:**
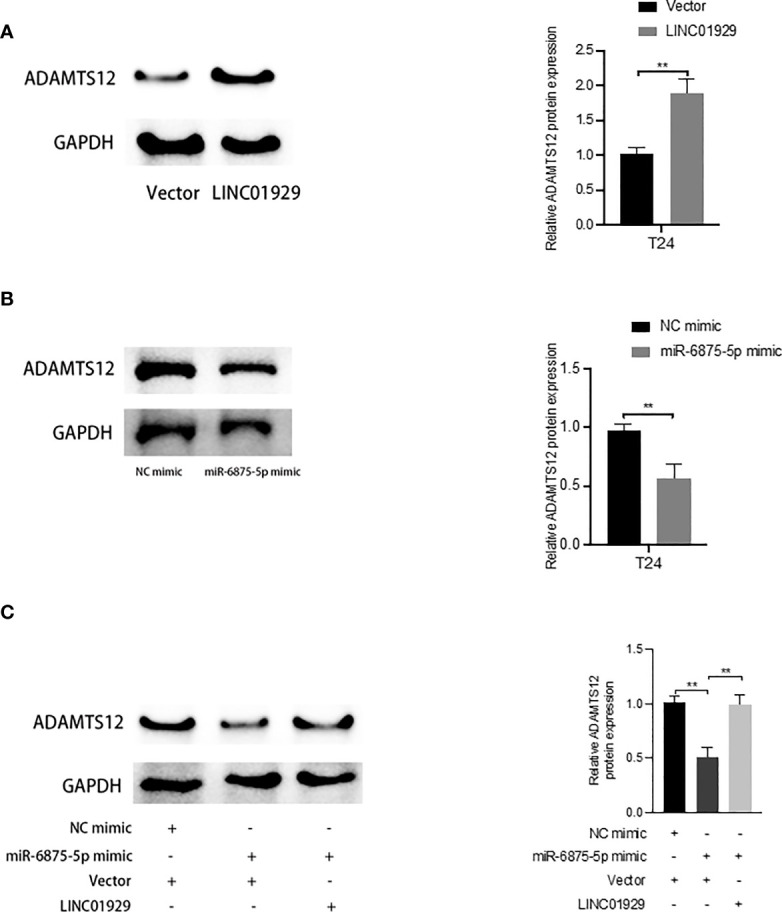
LINC01929 antagonizes miR-6875-5p mediated repression of ADAMTS12 expression. **(A, B)** The effect of LIN01929 and miR-6875-5p overexpression on ADAMTS12 protein expression levels in T24 cells was assessed by Western blotting. **(C)** Western blotting analysis of the effect of overexpressed LIN01929 on ADAMTS12 expression in T24 cells induced by miR-4875-5p overexpression. **P < 0.01.

To determine whether LINC01929 functions as a miRNA sponge in T24 cells. The luciferase reporter gene assay showed that miR-6875-5p overexpression significantly reduced the luciferase activity of LINC01929 compared to the NC group (**Figure 10B**). (**Figures 10A, B**) The failure of miR-6875-5p to affect the luciferase activity of LINC01929 after targeted mutagenesis of the predicted complementary binding site on LINC01929 supports the sponge effect of LINC01929 by binding to miR-6875-5p on specific sequences. Furthermore, pull-down analysis using biotin-labeled miR-6875-5p and control showed that LINC01929 was captured by miR-6875-5p in T24 cells, validating the interaction between LINC01929 and miR-6875-5p (**Figure 10C**). As the interaction between LINC01929 and miR-6875-5p was determined, we further investigated whether miR-6875-5p has a relationship with ADAMTS12. Using the same approach, we found the same results (**Figures 10D–F**), that is, there is also an interaction between miR-6875-5p and ADAMTS12. We next investigated whether LINC01929 could act as a sponge for miR-6875-5p to induce ADAMTS12 expression. qRT-PCR revealed that LINC01929 overexpression increased ADAMTS12 levels (**Figure 10G**), while overexpression of miR-6875-5p decreased ADAMTS12 levels in T24 (**Figure 10H**). Importantly, miR-6875-5p overexpression downregulated ADAMTS12 levels in T24, while concomitant overexpression of LINC01929 significantly reversed this effect (**Figure 10I**). Western blotting also showed the same results out of qRT-PCR (**Figures 11A–C**). Overall, these findings suggest that LINC01929 antagonizes the inhibitory effect of miR-6875-5p on ADAMTS12 expression *via* sponge adsorption.

## Discussion

Bladder cancer is a highly malignant and rapidly progressing cancer. Recent studies have revealed the prognostic outcomes of patients with BCa, particularly MIBC, with only 5% and 48% 5-year overall survival (OS) reported for untreated and treated groups, respectively. Additionally, the 5-year distant metastasis-free survival rate for untreated and treated BCa patients were only 26% and 57%, respectively (37). A large number of recent reports investigating ceRNA regulatory networks among coding RNAs and ncRNAs, both of which are extremely complex and intertwined, have been conducted. These reports indicate that functional ncRNA expression models and variation are critical in bladder carcinoma *via* the ceRNA networks mechanisms (38). However, few reports have investigated the correlation between ceRNA networks and the development and poor prognosis of advanced BCa. To explore the complicated regulation of molecules associated with the invasion, progression, and metastasis of advanced BCa at the transcriptional level, we utilized integrated lncRNAs, miRNAs, and mRNAs extracted from TCGA and GEO databases to construct core ceRNA regulatory networks based on the co-expression pattern and variation of functionally crucial RNAs in BCa patients.

In the present study, we constructed a core co-expression ceRNA network containing 8 lncRNAs- 28 mRNAs pairs and 8 miRNAs- 28 mRNAs pairs that survived multi-layered data screening. Through DE analysis and WGCNA, differentially expressed lncRNAs-mRNA and miRNAs were identified, having similar expression patterns in ACS-BCa. Following GO and KEGG enrichment, co-expressed mRNAs were enriched in the PI3K/AKT signaling pathway, ECM-receptor interaction, extracellular matrix, tissue development, response to growth factor stimulus, cell adhesion, and vasculature development, which was associated with BCa invasion, progression, and metastasis. Through survival analysis, all of the chosen 8 lncRNAs and 28 mRNAs significantly induced a poor prognosis in ACS-BCa. After performing predictions of mRNA-miRNA targeting, correlations among 8 miRNAs- 28 mRNAs pairs containing 8 co-expressed miRNAs with similar expression patterns were verified using Pearson correlation analysis, to construct a more stable 8 lncRNAs- 28 mRNAs- 8 miRNAs network containing lncRNAs-mRNAs pairs. This ceRNA network was theoretically established in the “ceRNA hypothesis”, reporting that multiple lncRNAs function as ceRNAs to sponge miRNAs, thus further regulating the expression of mRNAs and their translational proteins in carcinogenesis and tumor progression regulation (13, 38, 39).

By analyzing the results of the ceRNA network, we found that rising-trend expressions in all co-expressed lncRNAs and mRNAs in ACS-BCa, compared with LCS-BCa, differed from down-trends or invariable RNA expression in LCS-BCa, compared with normal urothelium. With regards to similar patterned miRNA expression, different tendencies were present in normal urothelium and ACS-BCa, compared with LCS-BCa. With this in mind, parts of upregulated lncRNAs and mRNAs, and unchanged miRNAs, may play a positive, non-independent role, like an entire network of similar expression patterns, in regulating the progression of LCS- to ACS-BCa, but an irrelevant or passive role in the transition from normal urothelium to LCS-BCa; based on the conjecture: As the whole ceRNA network, lncRNA and mRNA overexpression induces the invasion, progression, and metastasis of ACS-BCa with poor prognosis, regardless of miRNA expression trends. During regulatory mechanisms in the ceRNA network, we concluded that expression correlations between miRNAs and mRNAs in BCa tissue were predominately negative, indicating that these regulated miRNAs negatively correlated with lncRNAs who shared similar expression patterns and tendencies to mRNAs; thus constructing the lncRNAs-miRNAs-mRNAs network as a whole, rather than as individual RNA axes participating in regulatory mechanisms during ACS-BCa development. Recent reports support the aforementioned conjecture that an abundance of most individual targets is insufficient to alter active miRNAs (13). In summary, further experimental validations are required.

We attempted to experimentally validate the above inferences using BCa patient samples divided into clinically and pathologically different groups through bioinformatics analysis; so as to demonstrate the effect of 8 RNA expression patterns, as well as advanced BCa tendency to invade, progress, and metastasize. By probing recent reports, we dissected conflicting qRT-PCR, WB, and IHC results as follows:

In view of the sampling process, DE analysis was performed on samples of serum-circulating microRNA profiles, while qRT-PCR was performed with tissue biopsy samples from BCa tumor loci. A key challenge was investigating the origin of these miRNAs, whether they arose from the primary tumor or metastatic lesions, as not all tumor loci having identical circulating molecules to reflect tumors (9). This may be a reasonable explanation as to why differences in hsa-miR-128-2-5p expression between DE analysis and qRT-PCR were observed.

Coincidently, a non-significant decrease in EJ, and an increase in COL6A1, in the 5637 cell line, compared with the SV-HUC-1 cell line, may be understood with the conjecture that collagen VI promotes tumor growth with expression variation in the ECM (40), likely promoting differences in expression between tumor tissue and tumor cell lines (41, 42). According to recent reports, E−cadherin adhesive junctions integrated with cellular contractility play a critical role in tissue dynamics (43), with a loss of E-cadherin reducing cancer cell proliferation and survival, circulating tumor cell numbers, seeding of cancer cells in distant organs, and metastatic outgrowth (44). Similarly, the DE of CDH11 between tumor and normal cell lines may be non-significant, unlike in tissues. In terms of the mechanisms and pathophysiological angle, these results imply that the variation in expression of these genes plays a non-significant role in the transition from normal bladder urothelium to LCS-BCa tissue with oncogenic signatures, such as PIK3CA, STAG2, and TP53 mutations (1). Thus, further research is required.

Two conjectures accounted for the conflicting IHC results. DE between ADAMTS12 at the transcriptional and translational levels had been neglected before experimental design; T24, EJ, and 5637 cell lines were recently applied in studies exploring the invasive, progressive, and metastatic capabilities of advanced BCa, suggesting that they could be viewed as high-grade BCa cells (ACS-BCa) (45–47). This also supported the inconsistent expression of CDH11 in 5637, and COL6A1 in EJ and 5637, cell lines.

Intriguingly, IHC results revealed that non-significant differences in COL6A1, CDH11, and ADAMTS12 expression were present in the transition from pathologically non-invasive to invasive groups, both clinically non-muscle-invasive (stage TaT1). Thus, considering the upregulated expression of proteins in ACS-BCa tissue, compared with LCS-BCa tissue, bioinformatics inference further supported that these proteins play a critical role in the development of clinically muscle-invasive, rather than non-muscle-invasive, BCa.

Taken together, the bioinformatics analysis of expression changes was preliminarily verified through three experiments. Of the 8 RNAs tested, all lncRNAs and mRNAs demonstrated the same expression tendencies in the progression of LCS- to ACS-BCa, which was in agreement with DE analysis. Furthermore, a majority of RNAs showed both similar expression patterns and tendencies in the transition from normal bladder urothelium to LCS-BCa. Therefore, through the up or downregulation of miRNAs *via* the lncRNA-miRNA-mRNA regulatory mechanism, we noticed that all mRNAs played an important role in the invasion, progression, and metastasis of BCa linked to similarly expressed lncRNAs. Furthermore, the whole ceRNA network was supposed to be highlighted in ACS-BCa. Moreover, in DE analysis, COL6A1, CDH11, and ADAMTS12 tended to show a more significant expression tendency in BCa tissue than in BCa cell lines. In other words, these genes may play a role in ECM-receptor interactions and ECM remodeling, potentially promoting the development of advanced BCa. Evidence has emerged supporting the conjecture that altered ECM regulator activity in cancer triggers pathological ECM remodeling and facilitates metastatic dissemination, thus making ECM regulators potential targets for cancer therapy (33). Changes in the cellular microenvironment through Yes-associated protein induced by stiffness of the ECM plays a critical role in BCa development (48). This identifies a novel research route investigating ECM-related protein pathways and provides potential new prognostic biomarkers for advanced BCa patients.

To further validate the interactions between mRNAs, lncRNAs and miRNAs, this study first performed a series of functional experiments to elucidate the regulatory function of ADAMTS12 in BCa cells, and knockdown of ADAMTS12 had a significant inhibitory effect on tumor cells, i.e., reduced proliferation, increased apoptosis and attenuated cell migration, which was consistent with the previous bioinformatics analysis Although the mechanism of ADAMTS in carcinogenesis has not been fully elucidated, some studies have identified altered expression of individual members of the ADAMTS family in different tissues of human cancers, It is hypothesized that ADAMTS plays a role in tumorigenesis and development similar to that of matrix metalloproteinases and has oncogenic properties. It has been suggested that ADAMTS12 may be a promoter of GI tumors, responsible for tumor microenvironmental status and tumor energy metabolic transition (49). Subsequently, we verified the effects of LINC01929, miR-6875-5p and ADAMTS12 on the invasive function of bladder cancer cells through a series of functional experiments. Overexpression of LINC01929 and ADAMTS12 enhanced cell invasion, while overexpression of miR-6785-5p had the opposite effect. lncRNA function may depend on its downstream binding molecules, such as ceRNA, which can regulate changes in target mRNA expression by competitively binding miRNA (50). In gastric cancer, lncRNA MT1JP binds miR-92a-3p to regulate FBXW7 expression, which in turn affects gastric cancer progression (51). LINC01087 is enhanced in breast cancer, and LINC01087 affects ROCK1 expression by sponging miR-335-5p, thereby affecting migration and invasion of breast cells (52). Therefore, we proposed the hypothesis that LINC01929 upregulates miR-6875-5p through sponge adsorption and upregulates the oncogenic molecule ADAMST12. And the subsequent luciferase reporter gene assay directly verified that miR-6875-5p was a target of LINC01929, and the RNA pull down assay further verified the interaction between miR-6875-5p and LINC01929.

There are limitations in this study. We recognized stratification of BCa patients *via* TNM staging in TCGA, classifying the T2 stage (stage II) as a LCS-BCa grouping, which differed from the classification of BCa patient samples in previous experiments. Additionally, there were low numbers of BCa clinical samples for experimental verification, and only 19 normal bladder samples from TCGA for bioinformatics analysis, which produced unconvincing results. In summary, this study identified a LINC01929/miR-6875-5p/ADAMTS12 regulatory axis highly associated with advanced bladder cancer development through a network of core lncRNAs-miRNAs-mRNAs and validation of clinical samples, and elucidated that LINC01929 upregulates miR-6875-5p *via* sponge adsorption and upregulates the pro-cancer molecule ADAMST12, which in turn promotes the progression, invasion, and metastasis of advanced bladder cancer. It contributes to a comprehensive insight into the complex regulatory mechanisms of ceRNAs associated with ACS-BCa. Provides novel targets for the prognosis and diagnosis of advanced bladder cancer.

## Data Availability Statement

The original contributions presented in the study are included in the article/supplementary material. Further inquiries can be directed to the corresponding authors.

## Ethics Statement

Written informed consent was obtained from the individual(s) for the publication of any potentially identifiable images or data included in this article.

## Author Contributions

MP, YX, and YD contributed to the conception and design of the work. MP, YX, XY, JY, and LW conducted the experiments. MP and YX analyzed data and wrote this manuscript. LW and XL revised and reviewed the manuscript. All authors contributed to final approval of the version to be published and agree to be accountable for all aspects of the work.

## Funding

This study was supported by the National Natural Science Foundation of China (No. 81972408), The Key Research and Development Program of Hubei Province (No. 2020BCB051).

## Conflict of Interest

The authors declare that the research was conducted in the absence of any commercial or financial relationships that could be construed as a potential conflict of interest.

## Publisher’s Note

All claims expressed in this article are solely those of the authors and do not necessarily represent those of their affiliated organizations, or those of the publisher, the editors and the reviewers. Any product that may be evaluated in this article, or claim that may be made by its manufacturer, is not guaranteed or endorsed by the publisher.

## Abbreviations

BCa, bladder cancer; lncRNAs, long non-coding RNAs; miRNAs, micro RNAs; ceRNA, competitive endogenous RNA; DE analysis, differential expression analysis; ACS, advanced clinical stage; LCS, low clinical stage; MIBC, Muscle-invasive bladder cancer; mRNA, messenger RNA; EMT, epithelial-mesenchymal transition status; TCGA, The Cancer Genome Atlas; qRT-PCR, real time quantitative PCR; IHC, immunohistochemistry; GDC, NCI Genomic Data Commons; WGCNA, weighted gene co-expression network analysis; TO, topological overlap; GO, Gene ontology enrichment; BP, biological process; CC, cell component; MF, molecular function; KEGG, Kyoto Encyclopedia of Genes and Genomes; PPI, protein-protein-interaction; UCSC, University of California Santa Cruz; GAPDH, glyceraldehyde-3-phosphate dehydrogenase; WB, Western Blotting; PVDF, polyvinylidene difluoride; TBS-T, Tris-buffered saline with 0.05% Tween-20; ECL, enhanced chemiluminescence; siRNA, Small interfering RNA; cck-8, Cell Counting Kit-8 assay; PFA, paraformaldehyde; FDR, false discovery rate; PI3K, PI-3 kinase; CAFs, cancer-associated fibroblasts; ECM, extracellular matrix; OS, overall survival.
